# Inflammatory burden index predicts long-term mortality: evidence from population-based cohorts with clinical validation

**DOI:** 10.3389/fonc.2026.1932811

**Published:** 2026-10-15

**Authors:** Yuzheng Zhang, Xinyi Yu, Zhifang Jia, Shuo Yang, Wanying Huo, Chenhao Fu, Jing Jiang

**Affiliations:** 1 Department of Hospital Infection Management, China-Japan Union Hospital of Jilin University, Changchun, China; 2 Meihe Health Research Institute, The First Hospital of Jilin University, Changchun, China; 3 Department of Epidemiology and Biostatistics, School of Public Health, Jilin University, Changchun, China; 4 Division of Clinical Epidemiology, The First Hospital of Jilin University, Changchun, China

**Keywords:** cancer history, inflammatory burden index (IBI), mortality, population-based cohort, systemic inflammation

## Abstract

**Background:**

Systemic inflammation is increasingly recognized as a key determinant of long-term health outcomes. The Inflammatory Burden Index (IBI), derived from C-reactive protein, neutrophil count, and lymphocyte count, integrates multiple components of systemic inflammatory and immune status. However, evidence regarding its association with long-term mortality among individuals with and without a history of cancer remains limited.

**Methods:**

We investigated the associations of IBI with mortality outcomes using two population-based cohorts, the UK Biobank (UKB) and the National Health and Nutrition Examination Survey (NHANES, 1999–2010), and an independent clinical cohort of patients with gastric cancer. Multivariable Cox proportional hazards models were used to evaluate the associations between IBI and mortality outcomes. Stratified analyses were performed by history of cancer and among participants with digestive tract cancers, and sensitivity analyses were conducted to assess robustness.

**Results:**

The UKB included 455,423 participants, of whom 54,813 had a history of cancer, and NHANES included 24,708 participants, of whom 2,159 had a history of cancer. Participants with a history of cancer were more frequently represented in the highest IBI quartile in both cohorts. Higher IBI was consistently associated with increased all-cause, cancer-specific, and CVD-specific mortality. In the UKB, among participants with a history of cancer, the highest versus lowest IBI quartile was associated with higher risks of all-cause (HR = 1.62, 95% CI: 1.53–1.71), cancer-specific (HR = 1.55, 95% CI: 1.45–1.64), and CVD-specific mortality (HR = 1.81, 95% CI: 1.41–2.33). Consistent associations were observed in NHANES and remained robust in continuous and sensitivity analyses. Among participants with digestive tract cancers, higher IBI was associated with increased all-cause and cancer-specific mortality. In the independent gastric cancer cohort (n = 793), the highest IBI quartile was associated with poorer overall survival after multivariable adjustment (HR = 2.53, 95% CI: 1.59–4.02).

**Conclusion:**

Higher IBI was consistently associated with increased all-cause, cancer-specific, and CVD-specific mortality across population-based cohorts, with additional evidence from participants with digestive tract cancers and an independent gastric cancer cohort. These findings support the potential prognostic relevance of IBI as a readily accessible marker of systemic inflammatory burden.

## 1 Introduction

Chronic systemic inflammation is a recognized driver of cancer, cardiovascular disease and other chronic disorders (1, 2). In population-based studies, accurately quantifying individual inflammatory burden and identifying high-risk subgroups remain critical challenges. Conventional hematological indices derived from routine blood tests—such as neutrophil-to-lymphocyte ratio (NLR), platelet-to-lymphocyte ratio (PLR), lymphocyte-to-monocyte ratio (LMR), and systemic immune-inflammation index (SII)—have been extensively studied as surrogate markers of systemic inflammation (3, 4). Although these metrics correlate moderately with disease incidence and mortality, they capture only isolated aspects of the inflammatory response and often demonstrate inconsistent predictive performance across diverse populations (5).

The recently proposed inflammatory burden index (IBI) offers a potential solution to some of these limitations by integrating neutrophil count, lymphocyte count, and C-reactive protein (CRP) into a composite biomarker (6). By combining cellular immune parameters with an acute-phase reactant, IBI provides a more comprehensive reflection of systemic inflammatory–immune dysregulation, while retaining cost-effectiveness and accessibility through routine laboratory measurements. Emerging evidence suggests that elevated IBI is associated with increased risks of various health issues, including cardiovascular disease (7, 8), osteoarthritis (9), depression (10), increased all-cause mortality among older adults (11), and adverse outcomes in other disease-specific populations (12). These findings have prompted increasing interest in the potential prognostic relevance of IBI across different clinical and population settings.

In oncology, systemic inflammation plays a crucial role in tumor initiation, progression, and therapeutic response, interacting with local tumor microenvironment factors such as tumor-infiltrating lymphocytes (13). It has also been recognized as a hallmark of cancer, reflecting host–tumor immune dysregulation (14). Clinically, elevated preoperative IBI levels have been associated with adverse features including advanced tumor stage, malnutrition, postoperative complications, and poorer survival in patients with gastrointestinal and lung malignancies (15–19). Digestive tract cancers, which often arise from chronic inflammatory conditions (e.g., Helicobacter pylori-induced gastritis), serve as biologically relevant models for studying inflammation-related biomarkers (20). Nevertheless, evidence regarding the association between IBI and long-term mortality across both cancer and non-cancer populations remains limited, and its prognostic relevance in population-based cancer populations requires further investigation.

To address these gaps, we conducted a comprehensive investigation using two large population-based cohorts—the UK Biobank (UKB) and the National Health and Nutrition Examination Survey (NHANES)—to examine the associations of IBI with all-cause, cancer-specific, and cardiovascular disease (CVD)-specific mortality among participants with and without a history of cancer. We further evaluated these associations among participants with digestive tract malignancies and validated the findings in an independent clinical cohort of patients with gastric cancer undergoing curative gastrectomy. Through these complementary analyses, we aimed to evaluate the generalizability and prognostic relevance of IBI across population-based and oncological settings.

## 2 Material and methods

### 2.1 Study population

Discovery cohort: The UKB is a prospective cohort study comprising more than 500,000 participants aged 37–73 years who were recruited across the United Kingdom between 2006 and 2010. Ethical approval was granted by the North West Multi-Centre Research Ethics Committee, and all participants provided written informed consent (21). This study included individuals who completed the UKB baseline assessment during this recruitment period (n = 502,411). We excluded individuals with missing IBI components (n = 32,051), those who did not answer cancer-related questions (n = 2,616), and those with missing key covariates (n = 12,321), resulting in the final analytical cohort.

Validation cohort: The NHANES is a nationally representative, cross-sectional study conducted biennially using a complex, multistage probability sampling design to evaluate the health and nutritional status of the U.S. population. The National Center for Health Statistics Institutional Review Board granted ethical approval, and informed consent was obtained from all participants (22). Detailed methodology and survey protocols have been described previously (23). For this study, we included individuals who completed the NHANES questionnaire between 1999 and 2010 (n = 62,160). Participants who were younger than 20 years of age (n = 18,482) were excluded, as only adults were asked about cancer history. Individuals missing any variable required for IBI calculation (n = 14,934) or essential covariates (n = 4,036) were also excluded, yielding the final analytical sample.

Clinical validation cohort: An independent clinical cohort was used for external validation, comprising patients who underwent curative gastrectomy for gastric cancer at the First Hospital of Jilin University (Changchun, China) between 2011 and 2021. The following exclusion criteria were applied: (1) history of other malignancies or prior gastric surgery; (2) positive surgical margins; (3) incomplete laboratory data (missing CRP, neutrophil count, or lymphocyte count); (4) perioperative death; and (5) loss to follow-up at the first scheduled follow-up. Demographic, clinical, and pathological data were extracted from the institutional medical record system. All patients provided informed consent. This study was approved by the Ethics Committee of the First Hospital of Jilin University.

### 2.2 Assessment of inflammation burden levels

IBI quantifies the systemic inflammatory burden for each individual. IBI was calculated as standard CRP (mg/L) × neutrophil count (/μL)/lymphocyte count (/μL), according to the original definition (6). CRP concentrations were originally reported in mg/L in the UK Biobank and gastric cancer cohort and in mg/dL in NHANES. To harmonize CRP units across cohorts, NHANES CRP values were converted from mg/dL to mg/L by multiplying by 10 before IBI calculation. Thus, CRP was consistently expressed in mg/L for IBI calculation across all three cohorts. Laboratory measurements in the UK Biobank and NHANES were performed according to their standardized laboratory protocols. In NHANES, CRP values below the detection limit were handled according to the NHANES laboratory protocol by assigning the detection limit divided by the square root of two. In the gastric cancer cohort, hematological parameters were measured using an automated hematology analyzer (XE-2100, Sysmex, Japan), and CRP was measured using an automated biochemical analyzer (Beckman Coulter, USA) (24, 25). IBI was categorized into cohort-specific quartiles based on its distribution in each study population. The quartile cutoff values were 1.30, 2.82, and 6.35 in the UK Biobank; 1.35, 3.60, and 9.30 in NHANES; and 3.50, 9.02, and 46.05 in the gastric cancer cohort. In NHANES, quartiles were derived from the survey-weighted distribution. The overall cohort-specific quartile cutoffs were consistently applied to subgroup and sensitivity analyses.

#### 2.2.1 Ascertainment of mortality

The primary outcomes were all-cause mortality, which was further classified into cancer deaths and cardiovascular disease deaths based on ICD-10 codes, with cancer deaths classified using C00–C97 and CVD deaths classified using I00-I99. In the UKB, mortality data was obtained via linkage with national death registries, including NHS Digital for England and Wales and the NHS Central Register for Scotland. Mortality data were updated through April 2024.

In NHANES, mortality status was determined through linkage with the National Death Index, with follow-up available through December 31, 2019. Survival time was defined as the interval from the interview date to the date of death or end of follow-up, whichever came first.

For the gastric cancer cohort, overall survival (OS) was defined as the time from the date of surgery to the date of death, last successful follow-up, or the final follow-up date (December 2024), whichever occurred first. Follow-up procedures and data validation for this cohort have been described in detail in our previous study (26).

### 2.3 Covariates

Potential confounding factors were defined using comparable categories across the UK Biobank and NHANES cohorts where possible. The classification of variables was based on previous literature. Demographic variables encompassed age (categorized as <65 or ≥65 years), sex, race/ethnicity (non-Hispanic White vs. non-White in NHANES; White vs. other ethnicities in UKB), and body mass index (BMI), classified as underweight/normal (≤24.9 kg/m²), overweight (25–29.9 kg/m²), or obese (≥30 kg/m²). Socioeconomic factors included educational attainment (dichotomized as <high school or ≥high school), income status (NHANES: poverty-income ratio (15) categorized as low [≤ 1.5], medium [1.5–3.5], or high [>3.5]; UKB: pre-tax household income stratified as low [<£18,000], medium [£18,000–£100,000], or high [>£100,000]), and marital status (NHANES only: single [never married, widowed/divorced/separated] vs. in a relationship [married/living with partner]). Lifestyle factors comprised smoking (NHANES: ≥100 lifetime cigarettes vs. non-smokers; UKB: never vs. current/former smokers) and alcohol consumption (NHANES: ≥12 drinks/year vs. less; UKB: never vs. any intake). Clinical history included cancer, hypertension, diabetes, and cardiovascular diseases. For the gastric cancer cohort, clinical covariates included age, sex, tumor size, TNM stage, vascular invasion, neural invasion, WHO histological classification, histological differentiation, and chemotherapy.

### 2.4 Statistical analyses

In the UKB and gastric cancer cohorts, baseline characteristics were presented as counts and percentages. For NHANES, survey-specific sample weights were applied to account for the complex multistage, stratified sampling design, nonresponse, and post-stratification. Twelve-year combined weights were calculated across six survey cycles (1999–2010), and participant characteristics were reported as unweighted counts with weighted percentages. Distributions of categorical variables were compared using the chi-square test or Cochran–Armitage trend test, as appropriate. Associations between IBI and mortality outcomes were evaluated using Cox proportional hazards regression models, with hazard ratios (HRs) and 95% confidence intervals (CIs) reported. Multivariable models were adjusted for age, sex, race/ethnicity, BMI, education level, alcohol use, smoking status, income level, hypertension, and diabetes. Marital status was additionally adjusted for in NHANES. In the gastric cancer cohort, multivariable models were adjusted for age, sex, tumor size, TNM stage, vascular invasion, neural invasion, WHO histological classification, histological differentiation, and chemotherapy. Tests for trend across IBI quartiles were performed by modeling IBI quartiles as an ordinal variable. In addition to the quartile-based analyses, IBI was analyzed as a continuous variable. Given its right-skewed distribution, IBI was natural log-transformed and standardized, and HRs were estimated per 1-standard deviation (SD) increase in ln-transformed IBI. Multiplicative interactions between IBI quartiles and cancer status were evaluated by including interaction terms in the fully adjusted Cox models, with P values for interaction obtained from joint tests of the interaction terms. In the gastric cancer cohort, the Kaplan–Meier method was used to estimate 5-year overall survival and corresponding 95% CIs. Several sensitivity analyses were conducted to assess the robustness of the findings. To reduce the potential influence of reverse causation, analyses were repeated after excluding deaths occurring within the first 12 and 24 months of follow-up in the UK Biobank and NHANES cohorts, as well as among participants with digestive tract cancers in the UK Biobank. In addition, because markedly elevated CRP concentrations may reflect acute or clinically significant inflammatory states, analyses were repeated after excluding participants with CRP concentrations >10 mg/L in the UK Biobank, NHANES, and gastric cancer cohorts. The numbers of participants excluded and the final analytical sample sizes for each sensitivity analysis are reported in the **Supplementary Material**. Statistical analyses were performed using SPSS version 24.0 (IBM Corp., Armonk, NY, USA) and R version 4.5.0. All tests were two-sided, and *P* < 0.05 was considered statistically significant.

## 3 Results

### 3.1 Baseline characteristics of study participants

A total of 455,423 eligible participants were included in the UK Biobank cohort, including 54,813 participants with a history of cancer and 400,610 participants without a history of cancer. The NHANES cohort included 24,708 participants, of whom 2,159 had a history of cancer and 22,549 did not (**Figure 1**). **Table 1** presents the distribution of participants across IBI quartiles in the UK Biobank and NHANES cohorts. Participants with a history of cancer were more frequently represented in the higher IBI quartiles than those without a history of cancer (*P* < 0.001 in both cohorts). In the UK Biobank, 30.1% of participants with a history of cancer were in Q4, compared with 24.3% of those without a history of cancer. A similar pattern was observed in NHANES (30.6% vs. 24.5%).

**Figure 1:**
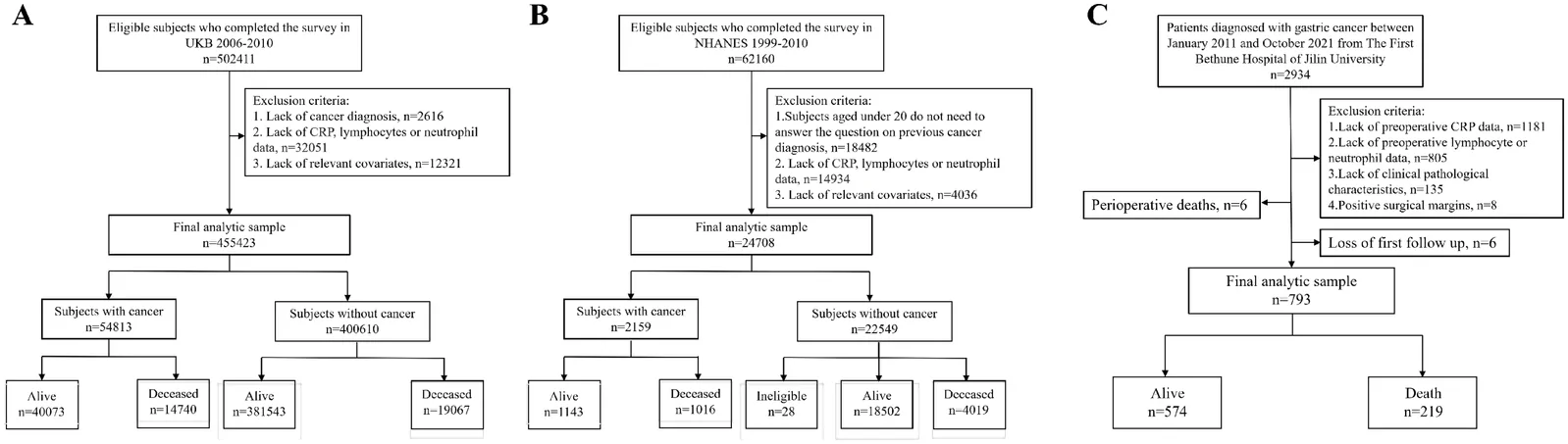
Flow chart of participant selection. **(A)** UKB cohort. **(B)** NHANES. **(C)** GC cohorts.

**Table 1:** Distribution of participants across IBI quartiles in the UKB and NHANES cohorts.

Characteristic	UKB			NHANES		
	Participants with a history of cancer	Participants without a history of cancer	*P* value	Participants with a history of cancer	Participants without a history of cancer	*P* value
IBI quartile			<0.001			<0.001
Q1	11352 (20.7)	102495 (25.6)		360 (19.4)	5126 (25.5)	
Q2	12875 (23.5)	101013 (25.2)		477 (22.8)	5440 (25.2)	
Q3	14093 (25.7)	99738 (24.9)		598 (27.2)	5682 (24.9)	
Q4	16493 (30.1)	97364 (24.3)		724 (30.6)	6301 (24.5)	

Detailed baseline characteristics stratified by cancer status are summarized in **Supplementary Table S.1**. In both cohorts, participants with a history of cancer were more likely to be older, female, and of White race/ethnicity than those without a history of cancer (all *P* < 0.001). They also had a higher prevalence of comorbidities, including diabetes and cardiovascular disease (all *P* < 0.001). In the UK Biobank, differences were observed in several sociodemographic characteristics, with participants with a history of cancer having lower educational attainment, lower income, and higher BMI than those without a history of cancer (all *P* ≤ 0.001). In NHANES, education level (*P* = 0.927) and BMI distribution (*P* = 0.545) did not differ significantly by cancer status, whereas differences were observed in marital status and income level (*P* = 0.035 and *P* = 0.040, respectively). Smoking and alcohol consumption did not differ significantly by cancer status in the UK Biobank but were more prevalent among participants with a history of cancer in NHANES (both *P* < 0.001).

### 3.2 Association between higher IBI and mortality risk in the UK biobank

As shown in **Table 2**, higher IBI levels were significantly associated with increased mortality risk in the UK Biobank. During follow-up, a total of 33,807 all-cause deaths were recorded, including 17,043 cancer-specific deaths and 6,926 CVD-specific deaths. A graded increase in mortality risk was observed across increasing IBI quartiles (all *P* for trend < 0.001). After multivariable adjustment, participants in Q4 had higher risks of all-cause (HR = 2.07, 95% CI: 2.00–2.15), cancer-specific (HR = 1.88, 95% CI: 1.78–1.98), and CVD-specific mortality (HR = 2.38, 95% CI: 2.18–2.61) compared with those in Q1.

**Table 2:** Associations of IBI with all-cause, cancer-specific, and CVD-specific mortality in the UK Biobank and NHANES cohorts.

Characteristics	IBI score (quartiles)				*P* trend
	Q1	Q2	Q3	Q4	
UKB Participants, n (%)	113847 (25.0)	113888 (25.0)	113831 (25.0)	113857 (25.0)	
All-cause mortality					
Events (n, %)	5227 (4.6)	6755 (5.9)	8443 (7.4)	13382 (11.8)	<0.001
Crude HR (95% CI)	1.00	1.30 (1.25-1.35)	1.64 (1.58-1.69)	2.66 (2.57-2.74)	<0.001
Adjusted HR (95% CI)	1.00	1.16 (1.12-1.21)	1.37 (1.32-1.43)	2.07 (2.00-2.15)	<0.001
Cancer-specific mortality					
Events (n, %)	2812 (2.5)	3560 (3.1)	4402 (3.9)	6269 (5.5)	<0.001
Crude HR (95% CI)	1.00	1.27 (1.21-1.33)	1.57 (1.50-1.65)	2.26 (2.16-2.36)	<0.001
Adjusted HR (95% CI)	1.00	1.18 (1.12-1.25)	1.38 (1.31-1.46)	1.88 (1.78-1.98)	<0.001
CVD-specific mortality					
Events (n, %)	864 (0.8)	1354 (1.2)	1685 (1.5)	3023 (2.7)	<0.001
Crude HR (95% CI)	1.00	1.57 (1.44-1.71)	1.96 (1.80-2.12)	3.52 (3.27-3.80)	<0.001
Adjusted HR (95% CI)	1.00	1.27 (1.16-1.40)	1.48 (1.34-1.62)	2.38 (2.18-2.61)	<0.001
NHANES Participants, n (weighted %)	5486 (25.0)	5917 (25.0)	6280 (25.1)	7025 (25.0)	
All-cause mortality					
Events (n, weighted %)	651 (7.8)	1085 (12.8)	1407 (16.4)	1892 (21.7)	<0.001
Crude HR (95% CI)	1.00	1.65 (1.46-1.87)	2.16 (1.91-2.43)	2.94 (2.62-3.29)	<0.001
Adjusted HR (95% CI)	1.00	1.23 (1.06-1.42)	1.54 (1.34-1.78)	2.06 (1.78-2.38)	<0.001
Cancer-specific mortality					
Events (n, weighted %)	172 (2.2)	231 (2.8)	332 (4.4)	410 (4.7)	<0.001
Crude HR (95% CI)	1.00	1.33 (1.04-1.71)	2.09 (1.65-2.65)	2.31 (1.83-2.90)	<0.001
Adjusted HR (95% CI)	1.00	0.98 (0.72-1.33)	1.48 (1.10-1.97)	1.65 (1.23-2.22)	<0.001
CVD-specific mortality					
Events (n, weighted %)	190 (2.3)	336 (3.6)	454 (5.1)	588 (6.3)	<0.001
Crude HR (95% CI)	1.00	1.56 (1.25-1.94)	2.11 (1.79-2.73)	2.86 (2.33-3.50)	<0.001
Adjusted HR (95% CI)	1.00	1.19 (0.90-1.56)	1.63 (1.25-2.12)	2.01 (1.55-2.61)	<0.001

Adjusted for age, sex, race/ethnicity, BMI, education level, alcohol use, smoking status, income level, hypertension, diabetes, and marital status (NHANES only).

Mortality was higher among participants with a history of cancer than among participants without a history of cancer (26.9% vs. 4.7%, *P* < 0.001; **Table 3**). After multivariable adjustment, higher IBI remained associated with increased mortality in both groups. Among participants with a history of cancer, those in Q4 had higher risks of all-cause (HR = 1.62, 95% CI: 1.53–1.71), cancer-specific (HR = 1.55, 95% CI: 1.45–1.64), and CVD-specific mortality (HR = 1.81, 95% CI: 1.41–2.33) compared with those in Q1. Among participants without a history of cancer, the corresponding HRs were 2.19 (95% CI: 2.09–2.31) for all-cause mortality, 1.84 (95% CI: 1.67–2.04) for cancer-specific mortality, and 2.50 (95% CI: 2.27–2.75) for CVD-specific mortality (all *P* for trend < 0.001). The corresponding cumulative incidence functions for cancer-specific and CVD-specific mortality, accounting for competing events, are presented in **Supplementary Figure S.1**. Sensitivity analyses yielded results consistent with the primary findings after excluding 924 participants who died within the first 12 months of follow-up (final n = 454,499), 2,297 participants who died within the first 24 months (final n = 453,126), and 18,797 participants with CRP concentrations >10 mg/L (final n = 436,626) (**Supplementary Tables S.2**-**S.5**). Formal interaction analyses indicated significant interactions between IBI and cancer status for all-cause, cancer-specific, and CVD-specific mortality (*P* for interaction < 0.001, < 0.001, and 0.035, respectively; **Supplementary Table S.6**), indicating that the magnitude of these associations differed according to cancer status in the UK Biobank. Consistent associations were also observed when IBI was modeled as a continuous variable, with higher IBI associated with increased all-cause, cancer-specific, and CVD-specific mortality (all *P* < 0.001; **Supplementary Table S.7**).

**Table 3:** Associations of IBI with all-cause, cancer-specific, and CVD-specific mortality stratified by cancer history.

Characteristics	IBI score (quartiles)				*P* trend
	Q1	Q2	Q3	Q4	
UKB: history of cancer, Participants n (%)	11352 (20.7)	12875 (23.5)	14093 (25.7)	16493 (30.1)	
All-cause mortality					
Events (n, %)	2343 (20.6)	3063 (23.8)	3733 (26.8)	5601 (34.0)	<0.001
Crude HR (95% CI)	1.00	1.17 (1.11-1.24)	1.33 (1.26-1.40)	1.81 (1.73-1.90)	<0.001
Adjusted HR (95% CI)	1.00	1.12 (1.06-1.19)	1.24 (1.17-1.32)	1.62 (1.53-1.71)	<0.001
Cancer-specific mortality					
Events (n, %)	2011 (17.7)	2614 (20.3)	3179 (22.6)	4571 (27.7)	<0.001
Crude HR (95% CI)	1.00	1.16 (1.10-1.23)	1.31 (1.24-1.39)	1.68 (1.60-1.77)	<0.001
Adjusted HR (95% CI)	1.00	1.14 (1.07-1.21)	1.25 (1.17-1.33)	1.55 (1.45-1.64)	<0.001
CVD-specific mortality					
Events (n, %)	106 (0.9)	171 (1.2)	213 (1.5)	383 (2.3)	<0.001
Crude HR (95% CI)	1.00	1.42 (1.12-1.81)	1.62 (1.28-2.04)	2.49 (2.01-3.09)	<0.001
Adjusted HR (95% CI)	1.00	1.20 (0.92-1.58)	1.27 (0.97-1.65)	1.81 (1.41-2.33)	<0.001
UKB: no history of cancer, Participants n (%)	102495 (25.6)	101013 (25.2)	99738 (24.9)	97364 (24.3)	
All-cause mortality					
Events (n, %)	2884 (2.8)	3692 (3.7)	4710 (4.7)	7781 (8.0)	<0.001
Crude HR (95% CI)	1.00	1.30 (1.24-1.37)	1.69 (1.61-1.77)	2.91 (2.78-3.03)	<0.001
Adjusted HR (95% CI)	1.00	1.14 (1.08-1.21)	1.38 (1.31-1.46)	2.19 (2.09-2.31)	<0.001
Cancer-specific mortality					
Events (n, %)	801 (0.8)	946 (0.9)	1223 (1.2)	1698 (1.7)	<0.001
Crude HR (95% CI)	1.00	1.20 (1.09-1.31)	1.57 (1.43-1.71)	2.23 (2.05-2.42)	<0.001
Adjusted HR (95% CI)	1.00	1.11 (1.00-1.23)	1.37 (1.23-1.51)	1.84 (1.67-2.04)	<0.001
CVD-specific mortality					
Events (n, %)	758 (0.7)	1183 (1.2)	1472 (1.5)	2640 (2.7)	<0.001
Crude HR (95% CI)	1.00	1.59 (1.45-1.74)	2.00 (1.83-2.19)	3.70 (3.41-4.01)	<0.001
Adjusted HR (95% CI)	1.00	1.28 (1.16-1.42)	1.51 (1.36-1.67)	2.50 (2.27-2.75)	<0.001
NHANES: history of cancer, Participants (n, weighted %)	360 (19.4)	477 (22.8)	598 (27.2)	724 (30.6)	
All-cause mortality					
Events (n, weighted %)	119 (4.2)	191 (7.0)	293 (10.7)	413 (15.2)	<0.001
Crude HR (95% CI)	1.00	1.47 (1.11-1.96)	2.01 (1.54-2.63)	2.81 (2.17-3.62)	<0.001
Adjusted HR (95% CI)	1.00	1.28 (0.96-1.70)	1.76 (1.35-2.31)	2.21 (1.69-2.89)	<0.001
Cancer-specific mortality					
Events (n, weighted %)	37 (1.3)	44 (1.6)	87 (3.4)	136 (4.7)	<0.001
Crude HR (95% CI)	1.00	1.00 (0.60-1.68)	1.89 (1.20-2.98)	2.36 (1.53-3.64)	<0.001
Adjusted HR (95% CI)	1.00	1.04 (0.57-1.89)	1.88 (1.10-3.22)	2.42 (1.43-4.10)	<0.001
CVD-specific mortality					
Events (n, weighted %)	27 (1.0)	65 (2.2)	79 (2.8)	99 (3.5)	<0.001
Crude HR (95% CI)	1.00	1.91 (1.14-3.18)	1.97 (1.20-3.25)	2.27 (1.40-3.69)	<0.001
Adjusted HR (95% CI)	1.00	1.57 (0.92-2.68)	1.63 (0.97-2.74)	1.61 (0.95-2.71)	0.134
NHANES: no history of cancer, Participants (n, weighted %)	5126 (25.5)	5440 (25.2)	5682 (24.9)	6301 (24.5)	
All-cause mortality					
Events (n, weighted %)	532 (1.8)	894 (2.9)	1114 (3.5)	1479 (4.6)	<0.001
Crude HR (95% CI)	1.00	1.66 (1.44-1.90)	2.09 (1.83-2.39)	2.80 (2.47-3.19)	<0.001
Adjusted HR (95% CI)	1.00	1.26 (1.07-1.48)	1.53 (1.30-1.80)	1.96 (1.66-2.30)	<0.001
Cancer-specific mortality					
Events (n, weighted %)	135 (0.5)	187 (0.6)	245 (0.9)	274 (0.9)	<0.001
Crude HR (95% CI)	1.00	1.35 (1.01-1.81)	1.93 (1.47-2.54)	1.76 (1.42-2.44)	<0.001
Adjusted HR (95% CI)	1.00	1.06 (0.75-1.49)	1.44 (1.03-2.02)	1.31 (0.93-1.85)	0.038
CVD-specific mortality					
Events (n, weighted %)	163 (0.6)	271 (0.8)	375 (1.1)	489 (1.4)	<0.001
Crude HR (95% CI)	1.00	1.44 (1.13-1.84)	2.10 (1.66-2.64)	2.64 (2.11-3.30)	<0.001
Adjusted HR (95% CI)	1.00	1.08 (0.80-1.45)	1.46 (1.10-1.94)	1.68 (1.26-2.23)	<0.001

Adjusted for age, sex, race/ethnicity, BMI, education level, alcohol use, smoking status, income level, hypertension, diabetes, and marital status.

### 3.3 Validation of IBI–mortality associations in NHANES

To assess the generalizability of the findings, the associations between IBI and mortality were further evaluated in NHANES. Consistent with the UK Biobank findings, higher IBI levels were significantly associated with increased mortality risk (**Table 2**). After multivariable adjustment, participants in Q4 had higher risks of all-cause mortality (HR = 2.06, 95% CI: 1.78–2.38), cancer-specific mortality (HR = 1.65, 95% CI: 1.23–2.22), and CVD-specific mortality (HR = 2.01, 95% CI: 1.55–2.61) compared with those in Q1 (all *P* for trend < 0.001).

Stratified analyses by cancer status showed that higher IBI was associated with increased mortality risk in both participants with and without a history of cancer (**Table 3**). Sensitivity analyses yielded results consistent with the primary findings after excluding 219 participants who died within the first 12 months of follow-up (final n = 24,489), 495 participants who died within the first 24 months (final n = 24,213), and 2,633 participants with CRP concentrations >10 mg/L (final n = 22,075) (**Supplementary Tables S.8**-**S.10**). However, formal interaction analyses showed no significant interactions between IBI and cancer status for all-cause, cancer-specific, or CVD-specific mortality (*P* for interaction = 0.859, 0.203, and 0.473, respectively; **Supplementary Table S.11**). Consistent associations were also observed when IBI was modeled as a continuous variable, with higher IBI associated with increased all-cause, cancer-specific, and CVD-specific mortality (all *P* < 0.001; **Supplementary Table S.12**).

### 3.4 Higher IBI is associated with increased mortality risk in participants with digestive tract cancers in the UK biobank

Given the inflammation-related nature of digestive tract cancers, we further evaluated the association between IBI and mortality within this subgroup. A total of 9,688 participants with digestive tract cancers were identified in the UK Biobank (**Supplementary Table S.13**). Compared with participants with non-digestive cancers, those with digestive tract cancers were more frequently categorized into the highest IBI quartile (32.5% vs. 29.6%, *P* < 0.001; **Supplementary Table S.14**).

As shown in **Table 4**, among the 9,688 participants with digestive tract cancers, 4,444 all-cause deaths were recorded, including 3,999 cancer-specific deaths and 166 CVD-specific deaths. After multivariable adjustment, participants in Q4 had higher risks of all-cause mortality (HR = 1.31, 95% CI: 1.18–1.45) and cancer-specific mortality (HR = 1.26, 95% CI: 1.14–1.39) compared with those in Q1 (both *P* for trend < 0.001). In contrast, the association with CVD-specific mortality was not statistically significant (HR = 1.12, 95% CI: 0.66–1.88; *P* for trend = 0.203). After excluding 129 participants who died within the first 12 months of follow-up (final n = 9,559) and 347 participants who died within the first 24 months (final n = 9,341), the associations of higher IBI with all-cause and cancer-specific mortality remained generally consistent with the primary findings (**Supplementary Tables S.15** and **S.16**). Given the limited number of participants with digestive tract cancers in NHANES (n = 198) and the relatively small number of outcome events, further validation in this subgroup was limited by statistical power.

**Table 4:** Associations of IBI with all-cause, cancer-specific, and CVD-specific mortality among participants with digestive tract cancers in the UK Biobank.

Characteristics	IBI score (quartiles)				*P* trend
	Q1	Q2	Q3	Q4	
Participants, n (%)	1738 (17.9)	2250 (23.2)	2555 (26.4)	3145 (32.5)	
All-cause mortality					
Events (n, %)	716 (41.2)	989 (44.0)	1155 (45.2)	1584 (50.4)	<0.001
Crude HR (95% CI)	1.00	1.10 (1.00-1.22)	1.14 (1.04-1.25)	1.36 (1.25-1.49)	<0.001
Adjusted HR (95% CI)	1.00	1.09 (0.98-1.22)	1.10 (0.99-1.23)	1.31 (1.18-1.45)	<0.001
Cancer-specific mortality					
Events (n, %)	659 (37.9)	918 (40.8)	1050 (41.1)	1372 (43.6)	0.001
Crude HR (95% CI)	1.00	1.11 (1.01-1.23)	1.13 (1.02-1.24)	1.28 (1.17-1.40)	<0.001
Adjusted HR (95% CI)	1.00	1.13 (1.01-1.26)	1.11 (1.00-1.24)	1.26 (1.14-1.39)	<0.001
CVD-specific mortality					
Events (n, %)	25 (1.4)	27 (1.2)	44 (1.7)	70 (2.2)	0.027
Crude HR (95% CI)	1.00	0.87 (0.51-1.49)	1.25 (0.76-2.04)	1.74 (1.10-2.74)	0.002
Adjusted HR (95% CI)	1.00	0.61 (0.33-1.13)	0.86 (0.50-1.49)	1.12 (0.66-1.88)	0.203

Adjusted for age, sex, race/ethnicity, BMI, education level, alcohol use, smoking status, income level, hypertension, diabetes, and marital status (NHANES only).

### 3.5 Preoperative IBI Is associated with long-term mortality in patients with gastric cancer

To further evaluate the prognostic relevance of IBI in a clinical setting, we analyzed an independent cohort of 793 patients with gastric cancer who underwent curative gastrectomy. Most patients were male (72.0%) and younger than 65 years (59.0%), and 70.4% had advanced-stage disease (TNM stage III/IV). Additional baseline clinical characteristics are summarized in **Supplementary Table S.17**. By the end of follow-up on December 31, 2024, 219 deaths (27.6%) had occurred. The median follow-up time was 42 months (95% CI: 39–46 months).

The distribution of IBI according to clinical characteristics is presented in **Supplementary Table S.18**. IBI distribution differed significantly according to age, tumor size, and TNM stage (all *P* < 0.001). Patients younger than 65 years and those with tumors <5 cm were more frequently represented in the lower IBI quartiles, whereas patients with stage III/IV disease were more frequently represented in the higher IBI quartiles. Differences in IBI distribution were also observed according to sex and vascular invasion (*P* = 0.039 and *P* = 0.035, respectively).

Survival analysis showed that higher preoperative IBI was associated with poorer overall survival (**Table 5**). The 5-year overall survival rate decreased from 63.6% (95% CI: 53.1–78.9) in Q1 to 37.2% (95% CI: 28.4–46.0) in Q4. After multivariable adjustment, compared with Q1, the adjusted HRs for all-cause mortality were 1.15 (95% CI: 0.67–1.95) for Q2, 2.01 (95% CI: 1.25–3.26) for Q3, and 2.53 (95% CI: 1.59–4.02) for Q4. After excluding 249 patients with CRP concentrations >10 mg/L, 544 patients remained in the sensitivity analysis. The graded association between higher IBI and all-cause mortality remained evident (*P* for trend = 0.007; **Supplementary Table S.19**). Consistent results were also observed when IBI was modeled as a continuous variable, with higher IBI associated with increased all-cause mortality (*P* < 0.001; **Supplementary Table S.20**).

**Table 5:** Association between IBI quartiles and all-cause mortality among patients with gastric cancer.

Characteristics	IBI score (quartiles)				
	Q1	Q2	Q3	Q4	*P* trend
Participants, n (%)	198 (25.0)	199 (25.1)	198 (25.0)	198 (25.0)	
Events (n, %)	26 (13.1)	30 (15.1)	58 (29.3)	105 (53.0)	<0.001
Crude HR (95% CI)	1.00	1.28 (0.75-2.17)	2.41 (1.51-3.84)	3.30 (2.14-5.08)	<0.001
Adjusted HR (95% CI)	1.00	1.15 (0.67-1.95)	2.01 (1.25-3.26)	2.53 (1.59-4.02)	<0.001

Adjusted for age, sex, tumor size, TNM stage, vascular invasion, neural invasion, WHO histological classification, histological differentiation, and chemotherapy.

## 4 Discussion

In this study, higher IBI was consistently associated with increased risks of all-cause, cancer-specific, and CVD-specific mortality across multiple populations, including the general population, individuals with a history of cancer, and patients with digestive tract malignancies. These associations were initially observed in the large prospective UK Biobank cohort and were subsequently replicated in NHANES. Further analyses in participants with digestive tract cancers and an independent clinical cohort of patients with gastric cancer supported the association between elevated IBI and poorer long-term outcomes in cancer populations. The associations remained generally robust across sensitivity analyses, including the exclusion of early deaths and participants with markedly elevated CRP concentrations, and were also observed when IBI was modeled as a continuous variable. Collectively, these findings support IBI as a potential marker of systemic inflammatory burden associated with long-term mortality risk across population-based and oncological settings.

IBI may serve as an integrative marker of systemic inflammatory burden because it incorporates three complementary components of the inflammatory response: neutrophils, lymphocytes, and CRP. In contrast to indices based solely on circulating blood cell ratios, such as NLR or PLR, IBI additionally incorporates the acute-phase response reflected by CRP, thereby capturing multiple dimensions of systemic inflammation and immune dysregulation (6). Previous studies have associated elevated IBI with adverse clinical outcomes in several disease and cancer populations (7–12). Our findings extend this evidence to large population-based cohorts and show that the association between higher IBI and mortality is not restricted to individuals with a history of cancer.

Notably, although CRP units were harmonized before IBI calculation, the distributions and corresponding quartile cutoffs of IBI differed across the UK Biobank, NHANES, and gastric cancer cohorts. These differences may reflect heterogeneity in population characteristics, underlying disease burden, inflammatory profiles, and cohort-specific sampling structures. Accordingly, the present findings support the prognostic relevance of higher IBI within populations rather than a universal absolute IBI threshold applicable across populations. Further studies are needed to determine whether standardized or clinically transferable IBI cutoffs can be established across diverse populations.

The association between IBI and mortality was also evident among participants with digestive tract malignancies, which are closely linked to chronic inflammatory processes. Persistent inflammatory stimuli, including Helicobacter pylori infection in gastric carcinogenesis, can contribute to tumor initiation and progression through sustained immune activation and alterations in the tumor microenvironment (27, 28). Consistent with previous studies of IBI in cancer populations (6), higher IBI was associated with increased all-cause and cancer-specific mortality among participants with digestive tract cancers in the UK Biobank. This association was further supported by the independent gastric cancer cohort, in which higher preoperative IBI was associated with poorer overall survival after curative gastrectomy. Previous studies have similarly linked elevated IBI with postoperative complications, poorer survival, malnutrition, and cachexia in patients with cancer (19). Together, these findings suggest that IBI may provide additional information regarding systemic inflammatory status and prognosis in patients with gastrointestinal malignancies.

Given that IBI is derived from routinely available laboratory parameters, including CRP, neutrophil count, and lymphocyte count, it may represent a practical and readily accessible marker for mortality risk stratification. In clinical settings, particularly among patients undergoing surgery for gastric cancer, preoperative IBI may help identify individuals at higher risk of adverse long-term outcomes who could benefit from closer clinical surveillance and comprehensive assessment of inflammatory and nutritional status. However, whether IBI can provide incremental prognostic value beyond established clinical risk factors or guide perioperative and oncological management remains to be determined. Future prospective studies are warranted to validate its clinical utility and to evaluate whether incorporating IBI into existing risk-stratification frameworks can improve clinical decision-making.

Several biological mechanisms may underlie the observed association between elevated IBI and mortality, particularly cancer-related mortality. Neutrophil activation can promote tumor progression through the release of reactive oxygen species, proteases, and other inflammatory mediators, thereby contributing to DNA damage and genomic instability (29, 30). Neutrophil extracellular traps may further facilitate tumor growth and metastatic processes by sustaining a pro-tumor inflammatory microenvironment (31). Conversely, lymphopenia may reflect impaired adaptive antitumor immunity and reduced immune surveillance, including compromised cytotoxic T-cell and natural killer cell activity (32). Concurrently, elevated CRP levels—driven by tumor-derived cytokines such as interleukin-6 and tumor necrosis factor-alpha—enhance cancer aggressiveness by creating a proinflammatory niche that supports angiogenesis, metastasis, and therapy resistance (33). This cytokine-mediated CRP elevation not only reflects systemic inflammation but also actively contributes to a tumor-promoting microenvironment by suppressing antitumor immunity and enhancing extracellular matrix remodeling (34). Taken together, by capturing the interplay between neutrophil-driven DNA damage, lymphocyte-depleted immune dysfunction, and CRP-sustained inflammatory signaling, IBI may provide an integrated measure of systemic inflammatory and immune dysregulation associated with cancer mortality risk. These findings provide further support for the potential involvement of these inflammatory and immune pathways in the association between elevated IBI and mortality, although further mechanistic studies are warranted to elucidate the underlying biological processes.

Importantly, higher IBI was also consistently associated with CVD-specific mortality in both population-based cohorts, suggesting that the relevance of systemic inflammatory burden may extend beyond cancer-related outcomes. Several biological pathways may help explain this association. Chronic systemic inflammation is closely involved in endothelial dysfunction, atherosclerotic plaque progression, and thrombosis (35). Neutrophil activation may promote endothelial injury, oxidative stress, and thrombo-inflammatory responses, whereas relative lymphocyte depletion may reflect impaired adaptive immune regulation (36). Together with elevated CRP, these alterations may characterize a sustained inflammatory milieu that promotes vascular injury and plaque instability. Consistent with this interpretation, previous population-based studies have linked neutrophil-dominant inflammatory indices, including NLR and SIRI, to cardiovascular disease (37), while IBI itself has also been associated with CVD in US adults (38). Thus, our findings further support the potential relevance of IBI to long-term cardiovascular outcomes, although further studies are warranted to clarify the underlying mechanisms and its potential role in cardiovascular risk assessment.

The consistency of the findings across several complementary analyses further supports the robustness of the observed associations. Similar results were obtained after excluding deaths occurring within the first 12 and 24 months of follow-up, reducing the likelihood that the primary findings were driven entirely by severe underlying disease shortly before death. Associations also remained evident after excluding participants with markedly elevated CRP concentrations, suggesting that the findings were not solely attributable to individuals with pronounced inflammatory states at baseline. Moreover, analyses treating IBI as a continuous variable produced consistent results, indicating that the observed associations were not dependent on quartile categorization alone. Nevertheless, because CRP and circulating leukocyte counts are dynamic and can be influenced by infection, treatment, comorbidities, and other transient conditions, a single baseline IBI measurement cannot fully characterize long-term inflammatory burden.

The strengths of this study include the use of two large population-based cohorts, replication across independent study populations, assessment of multiple mortality outcomes, and additional evaluation in participants with digestive tract cancers and an independent gastric cancer cohort. The availability of detailed covariate information enabled adjustment for a broad range of potential confounders, while sensitivity analyses and continuous-variable analyses further supported the robustness of the main findings. However, several limitations should be acknowledged. First, the observational design precludes causal inference, and residual confounding from unmeasured or incompletely measured factors cannot be excluded. Although exclusion of early deaths reduced concerns regarding reverse causation, this possibility cannot be completely eliminated. Second, IBI was derived from a single baseline measurement, precluding assessment of longitudinal changes in inflammatory burden and their relationships with subsequent outcomes. Third, detailed information on cancer type, stage, treatment, and time since diagnosis was not uniformly available across the population-based cohorts, limiting more detailed evaluation of cancer-related heterogeneity. Fourth, some subgroup analyses, particularly those involving digestive tract cancers in NHANES, were constrained by limited sample sizes and outcome events. Fifth, multiple outcomes and subgroup analyses were examined without formal adjustment for multiple comparisons; therefore, subgroup findings should be interpreted with caution given the potential for type I error. Finally, although the association between IBI and mortality was replicated across several populations, its incremental predictive performance beyond established risk factors and its clinical utility have not yet been established. Further prospective studies incorporating repeated IBI measurements, detailed cancer-related characteristics, and formal evaluation of incremental predictive performance are warranted.

## 5 Conclusion

In conclusion, higher IBI was consistently associated with increased all-cause, cancer-specific, and CVD-specific mortality across the UK Biobank and NHANES cohorts, with additional evidence from participants with digestive tract cancers and an independent gastric cancer cohort. These findings support IBI as a readily accessible marker of systemic inflammatory burden with potential value for mortality risk stratification. Further prospective studies are needed to establish its incremental predictive value and clinical utility.

## Data Availability

The original contributions presented in the study are included in the article/**Supplementary Material**. Further inquiries can be directed to the corresponding author.
