# Supplementary material for: Inflammatory burden index predicts long-term mortality: evidence from population-based cohorts with clinical validation
Source: Front Oncol. 2026 Oct 15;16:1932811. doi: 10.3389/fonc.2026.1932811 (PMC13716599; doi:10.3389/fonc.2026.1932811)
Supplement: Supplementary file 1 [file SupplementaryFile1.docx]

Supplementary Material

# 1 Supplementary Figures and Tables

**Table S.1 General characteristics of study participants in the UKB and NHANES cohorts.**

| **Characteristic** | **UKB** | | | |  | **NHANES** | | | |
| --- | --- | --- | --- | --- | --- | --- | --- | --- | --- |
|  | All participants | Participants with a history of cancer | Participants without a history of cancer | *P* value |  | All participants | Participants with a history of cancer | Participants without a history of cancer | *P* value |
| **Age (years), mean (SD)** | 56.5 (8.1) | 60.0 (6.9) | 56.1 (8.1) | <0.001 |  | 45.8 (16.5) | 61.1 (15.4) | 44.4 (15.9) | <0.001 |
| **Age (years, n, %)** |  |  |  | <0.001 |  |  |  |  | <0.001 |
| <65 | 388528 (85.3) | 41912 (76.5) | 346616 (86.5) |  |  | 18767 (84.4) | 860 (52.7) | 17907 (87.2) |  |
| ≥65 | 66895 (14.7) | 12901 (23.5) | 53994 (13.5) |  |  | 5941 (15.6) | 1299 (47.3) | 4642 (12.8) |  |
| **Sex (n, %)** |  |  |  | <0.001 |  |  |  |  | <0.001 |
| Male | 209030 (45.9) | 26388 (48.1) | 182642 (45.6) |  |  | 12010 (48.8) | 1029 (41.6) | 10981 (49.4) |  |
| Female | 246393 (54.1) | 28425 (51.9) | 217968 (54.4) |  |  | 12698 (51.2) | 1130 (58.4) | 11568 (50.6) |  |
| **Race/ethnicity (n, %)** |  |  |  | <0.001 |  |  |  |  | <0.001 |
| White | 430389 (94.5) | 52688 (96.1) | 377701 (94.3) |  |  | 12463 (84.9) | 1642 (95.8) | 10821 (83.8) |  |
| Non-white | 25027 (5.5) | 2125 (3.9) | 22902 (5.7) |  |  | 6743 (15.1) | 224 (4.2) | 6519 (16.2) |  |
| **Education (n, %)** |  |  |  | <0.001 |  |  |  |  | 0.927 |
| <High school | 77405 (17.2) | 11750 (21.7) | 65655 (16.6) |  |  | 13212 (44.0) | 1093 (43.9) | 12119 (44.0) |  |
| ≥High school | 373410 (82.8) | 42430 (78.3) | 330980 (83.4) |  |  | 11466 (56.0) | 1064 (56.1) | 10402 (56.0) |  |
| **Marital status (n, %)** |  |  |  |  |  |  |  |  | 0.035 |
| Single | - | - | - |  |  | 9193 (34.9) | 770 (32.1) | 8423 (35.1) |  |
| In a relationship | - | - | - |  |  | 15102 (65.1) | 1371 (67.9) | 13731 (64.9) |  |
| **Income level (n, %)** |  |  |  | <0.001 |  |  |  |  | 0.040 |
| Low | 88834 (22.6) | 12877 (27.7) | 75957 (21.9) |  |  | 9835 (29.1) | 720 (26.4) | 9115 (29.4) |  |
| Middle | 282913 (72.0) | 31599 (68.0) | 251314 (72.5) |  |  | 7513 (30.1) | 719 (30.3) | 6794 (30.1) |  |
| High | 21297 (5.4) | 1960 (4.2) | 19337 (5.6) |  |  | 7360 (40.7) | 720 (43.3) | 6640 (40.5) |  |
| **BMI (kg/m^2^, n, %)** |  |  |  | 0.001 |  |  |  |  | 0.545 |
| ≤24.9 | 145850 (32.7) | 15913 (29.6) | 129937 (33.1) |  |  | 7280 (32.9) | 619 (31.8) | 6661 (33.0) |  |
| 25-29.9 | 190730 (42.7) | 23518 (43.7) | 167212 (42.6) |  |  | 8651 (34.5) | 772 (34.5) | 7879 (34.5) |  |
| ≥30.0 | 109939 (24.6) | 14339 (26.7) | 95600 (24.3) |  |  | 8410 (32.6) | 715 (33.7) | 7695 (32.5) |  |
| **Alcohol use (n, %)** |  |  |  | 0.467 |  |  |  |  | <0.001 |
| No | 36246 (8.0) | 4317 (7.9) | 31929 (8.0) |  |  | 16453 (74.8) | 1419 (69.8) | 15034 (75.2) |  |
| Yes | 418759 (92.0) | 50443 (92.1) | 368316 (92.0) |  |  | 6878 (25.2) | 660 (30.2) | 6218 (24.8) |  |
| **Tobacco use (n, %)** |  |  |  | 0.497 |  |  |  |  | <0.001 |
| No | 248435 (54.8) | 29891 (54.8) | 218544 (54.8) |  |  | 12906 (51.6) | 934 (42.9) | 11972 (52.4) |  |
| Yes | 205285 (45.2) | 24696 (45.2) | 180589 (45.2) |  |  | 11784 (48.4) | 1223 (57.1) | 10561 (47.6) |  |
| **Hypertension (n, %)** |  |  |  | 0.345 |  |  |  |  | <0.001 |
| No | 225251 (49.5) | 27274 (49.8) | 197977 (49.5) |  |  | 18899 (80.8) | 1440 (72.1) | 17459 (81.6) |  |
| Yes | 229684 (50.0) | 27479 (50.2) | 202205 (50.5) |  |  | 5809 (19.2) | 719 (27.9) | 5090 (18.4) |  |
| **Diabetes (n, %)** |  |  |  | <0.001 |  |  |  |  | <0.001 |
| No | 430687 (94.6) | 51048 (93.4) | 379639 (95.0) |  |  | 22131 (92.7) | 1809 (87.2) | 20322 (93.2) |  |
| Yes | 23704 (5.2) | 3635 (6.6) | 20069 (5.0) |  |  | 2577 (7.3) | 350 (12.8) | 2227 (6.8) |  |
| **CVD (n, %)** |  |  |  | <0.001 |  |  |  |  | <0.001 |
| No | 316928 (69.7) | 34771 (63.5) | 282157 (70.6) |  |  | 22190 (92.3) | 1650 (81.2) | 20540 (93.3) |  |
| Yes | 137626 (30.3) | 19945 (36.5) | 117681 (29.4) |  |  | 2518 (7.7) | 509 (18.8) | 2009 (6.7) |  |

**Table S.2 Sample sizes for the sensitivity analyses across the study populations.**

| **Sensitivity analysis** | **Participants excluded, n** | **Final analytical sample, n** |
| --- | --- | --- |
| **UK Biobank (n=455423)** |  |  |
| Exclusion of deaths occurring within the first 12 months of follow-up | 924 | 454499 |
| Exclusion of deaths occurring within the first 24 months of follow-up | 2,297 | 453126 |
| Exclusion of participants with CRP >10 mg/L | 18,797 | 436626 |
| **Participants with digestive tract cancers in the UK Biobank (n=9688)** |  |  |
| Exclusion of deaths occurring within the first 12 months of follow-up | 129 | 9559 |
| Exclusion of deaths occurring within the first 24 months of follow-up | 347 | 9341 |
| **NHANES (n=24708)** |  |  |
| Exclusion of deaths occurring within the first 12 months of follow-up | 219 | 24489 |
| Exclusion of deaths occurring within the first 24 months of follow-up | 495 | 24213 |
| Exclusion of participants with CRP >10 mg/L | 2,633 | 22075 |
| **Gastric cancer cohort (n=793)** |  |  |
| Exclusion of patients with CRP >10 mg/L | 249 | 544 |

**Table S.3** **Sensitivity analyses of the associations between IBI and mortality after excluding deaths occurring within the first 12 months of follow-up in the UK Biobank**

|  | **IBI score (quartiles)** | | | | ***P* trend** |
| --- | --- | --- | --- | --- | --- |
|  | **Q1** | **Q2** | **Q3** | **Q4** |  |
| **All participants (n, %)** | 113766 (25.0) | 113773 (25.0) | 113650 (25.0) | 113310 (25.0) |  |
| **All-cause mortality** |  |  |  |  |  |
| Events (n, %) | 5146 (4.5) | 6640 (5.8) | 8262 (7.3) | 12835 (11.3) | <0.001 |
| Crude HR (95% CI) | 1.00 | 1.30 (1.25-1.35) | 1.63 (1.57-1.68) | 2.59 (2.51-2.67) | <0.001 |
| Adjusted HR (95% CI) | 1.00 | 1.15 (1.11-1.20) | 1.36 (1.31-1.42) | 2.00 (1.93-2.08) | <0.001 |
| **Cancer-specific mortality** |  |  |  |  |  |
| Events (n, %) | 2782 (2.4) | 3512 (3.1) | 4331 (3.8) | 5948 (5.2) | <0.001 |
| Crude HR (95% CI) | 1.00 | 1.26 (1.20-1.33) | 1.57 (1.49-1.64) | 2.17 (2.08-2.27) | <0.001 |
| Adjusted HR (95% CI) | 1.00 | 1.10 (1.00-1.23) | 1.35 (1.22-1.50) | 1.79 (1.62-1.98) | <0.001 |
| **CVD-specific mortality** |  |  |  |  |  |
| Events (n, %) | 838 (0.7) | 1320 (1.2) | 1618 (1.4) | 2902 (2.6) | <0.001 |
| Crude HR (95% CI) | 1.00 | 1.58 (1.44-1.72) | 1.93 (1.78-2.10) | 3.49 (3.23-3.77) | <0.001 |
| Adjusted HR (95% CI) | 1.00 | 1.28 (1.16-1.43) | 1.49 (1.35-1.66) | 2.47 (2.24-2.72) | <0.001 |
| **Participants with a history of cancer, (n, %)** | 11327 (20.8) | 12834 (23.6) | 14031 (25.8) | 16213 (29.8) |  |
| **All-cause mortality** |  |  |  |  |  |
| Events (n, %) | 2318 (20.5) | 3022 (23.5) | 3671 (26.2) | 5321 (32.8) | <0.001 |
| Crude HR (95% CI) | 1.00 | 1.17 (1.11-1.24) | 1.33 (1.26-1.40) | 1.75 (1.66-1.83) | <0.001 |
| Adjusted HR (95% CI) | 1.00 | 1.12 (1.05-1.19) | 1.23 (1.16-1.30) | 1.54 (1.46-1.63) | <0.001 |
| **Cancer-specific mortality** |  |  |  |  |  |
| Events (n, %) | 1988 (17.6) | 2577 (20.1) | 3127 (22.3) | 4310 (26.6) | <0.001 |
| Crude HR (95% CI) | 1.00 | 1.16 (1.10-1.23) | 1.31 (1.24-1.38) | 1.61 (1.53-1.70) | <0.001 |
| Adjusted HR (95% CI) | 1.00 | 1.13 (1.06-1.20) | 1.23 (1.15-1.31) | 1.46 (1.37-1.55) | <0.001 |
| **CVD-specific mortality** |  |  |  |  |  |
| Events (n, %) | 106 (0.9) | 168 (1.3) | 207 (1.5) | 374 (2.3) | <0.001 |
| Crude HR (95% CI) | 1.00 | 1.40 (1.10-1.78) | 1.58 (1.25-1.99) | 2.47 (1.99-3.06) | <0.001 |
| Adjusted HR (95% CI) | 1.00 | 1.18 (0.90-1.55) | 1.25 (0.96-1.64) | 1.81 (1.40-2.32) | <0.001 |
| **Participants without a history of cancer (n, %)** | 102439 (25.6) | 100939 (25.2) | 99619 (24.9) | 97097 (24.3) |  |
| **All-cause mortality** |  |  |  |  |  |
| Events (n, %) | 2828 (2.8) | 3618 (3.6) | 4591 (4.6) | 7514 (7.7) | <0.001 |
| Crude HR (95% CI) | 1.00 | 1.30 (1.24-1.37) | 1.68 (1.60-1.76) | 2.86 (2.74-2.99) | <0.001 |
| Adjusted HR (95% CI) | 1.00 | 1.14 (1.07-1.20) | 1.37 (1.30-1.45) | 2.14 (2.04-2.26) | <0.001 |
| **Cancer-specific mortality** |  |  |  |  |  |
| Events (n, %) | 794 (0.8) | 935 (0.9) | 1204 (1.2) | 1638 (1.7) | <0.001 |
| Crude HR (95% CI) | 1.00 | 1.19 (1.09-1.31) | 1.56 (1.42-1.70) | 2.17 (2.00-2.37) | <0.001 |
| Adjusted HR (95% CI) | 1.00 | 1.10 (1.00-1.23) | 1.35 (1.22-1.50) | 1.79 (1.62-1.98) | <0.001 |
| **CVD-specific mortality** |  |  |  |  |  |
| Events (n, %) | 732 (0.7) | 1152 (1.1) | 1411 (1.4) | 2528 (2.6) | <0.001 |
| Crude HR (95% CI) | 1.00 | 1.60 (1.46-1.75) | 1.98 (1.81-2.17) | 3.66 (3.37-3.97) | <0.001 |
| Adjusted HR (95% CI) | 1.00 | 1.28 (1.16-1.43) | 1.49 (1.35-1.66) | 2.47 (2.24-2.72) | <0.001 |

Note: Adjusted for age, sex, race/ethnicity, BMI, education level, alcohol use, smoking status, income level, hypertension, and diabetes.

**Table S.4** **Sensitivity analyses of the associations between IBI and mortality after excluding deaths occurring within the first 24 months of follow-up in the UK Biobank**

|  | **IBI score (quartiles)** | | | | ***P* trend** |
| --- | --- | --- | --- | --- | --- |
|  | **Q1** | **Q2** | **Q3** | **Q4** |  |
| **All participants (n, %)** | 113602 (25.1) | 113559 (25.1) | 113318 (25.0) | 112647 (24.8) |  |
| **All-cause mortality** |  |  |  |  |  |
| Events (n, %) | 4982 (4.4) | 6426 (5.7) | 7930 (7.0) | 12172 (10.8) | <0.001 |
| Crude HR (95% CI) | 1.00 | 1.30 (1.25-1.34) | 1.61 (1.56-1.67) | 2.59 (2.46-2.62) | <0.001 |
| Adjusted HR (95% CI) | 1.00 | 1.15 (1.10-1.20) | 1.34 (1.29-1.40) | 1.95 (1.87-2.03) | <0.001 |
| **Cancer-specific mortality** |  |  |  |  |  |
| Events (n, %) | 2690 (2.4) | 3376 (3.0) | 4142 (3.7) | 5554 (4.9) | <0.001 |
| Crude HR (95% CI) | 1.00 | 1.26 (1.20-1.33) | 1.56 (1.49-1.64) | 2.14 (2.05-2.24) | <0.001 |
| Adjusted HR (95% CI) | 1.00 | 1.16 (1.09-1.23) | 1.35 (1.28-1.43) | 1.76 (1.66-1.86) | <0.001 |
| **CVD-specific mortality** |  |  |  |  |  |
| Events (n, %) | 809 (0.7) | 1273 (1.1) | 1528 (1.3) | 2765 (2.5) | <0.001 |
| Crude HR (95% CI) | 1.00 | 1.58 (1.45-1.73) | 1.91 (1.76-2.09) | 3.55 (3.29-3.84) | <0.001 |
| Adjusted HR (95% CI) | 1.00 | 1.27 (1.15-1.40) | 1.43 (1.30-1.57) | 2.39 (2.18-2.61) | <0.001 |
| **Participants with a history of cancer, (n, %)** | 11251 (21.0) | 12722 (23.7) | 13861 (25.8) | 15869 (29.5) |  |
| **All-cause mortality** |  |  |  |  |  |
| Events (n, %) | 2242 (19.9) | 2910 (22.9) | 3501 (25.3) | 4977 (31.4) | <0.001 |
| Crude HR (95% CI) | 1.00 | 1.17 (1.11-1.23) | 1.31 (1.24-1.38) | 1.69 (1.61-1.78) | <0.001 |
| Adjusted HR (95% CI) | 1.00 | 1.11 (1.04-1.18) | 1.19 (1.13-1.26) | 1.49 (1.41-1.58) | <0.001 |
| **Cancer-specific mortality** |  |  |  |  |  |
| Events (n, %) | 1916 (17.0) | 2473 (19.4) | 2974 (21.5) | 4000 (25.2) | <0.001 |
| Crude HR (95% CI) | 1.00 | 1.16 (1.09-1.23) | 1.30 (1.23-1.38) | 1.59 (1.50-1.67) | <0.001 |
| Adjusted HR (95% CI) | 1.00 | 1.09 (0.98-1.21) | 1.35 (1.21-1.50) | 1.79 (1.61-1.98) | <0.001 |
| **CVD-specific mortality** |  |  |  |  |  |
| Events (n, %) | 103 (0.9) | 164 (1.3) | 195 (1.4) | 362 (2.3) | <0.001 |
| Crude HR (95% CI) | 1.00 | 1.44 (1.12-1.84) | 1.60 (1.26-2.02) | 2.71 (2.18-3.38) | <0.001 |
| Adjusted HR (95% CI) | 1.00 | 1.20 (0.91-1.58) | 1.21 (0.92-1.58) | 1.94 (1.52-2.53) | <0.001 |
| **Participants without a history of cancer (n, %)** | 102351 (25.6) | 100837 (25.2) | 99457 (25.0) | 96778 (24.2) |  |
| **All-cause mortality** |  |  |  |  |  |
| Events (n, %) | 2740 (2.7) | 3516 (3.5) | 4429 (4.5) | 7195 (7.4) | <0.001 |
| Crude HR (95% CI) | 1.00 | 1.31 (1.24-1.37) | 1.67 (1.60-1.76) | 2.83 (2.71-2.96) | <0.001 |
| Adjusted HR (95% CI) | 1.00 | 1.14 (1.07-1.20) | 1.36 (1.28-1.43) | 2.12 (2.02-2.23) | <0.001 |
| **Cancer-specific mortality** |  |  |  |  |  |
| Events (n, %) | 774 (0.8) | 903 (0.9) | 1168 (1.2) | 1554 (1.6) | <0.001 |
| Crude HR (95% CI) | 1.00 | 1.19 (1.09-1.31) | 1.56 (1.43-1.71) | 2.17 (1.99-2.36) | <0.001 |
| Adjusted HR (95% CI) | 1.00 | 1.12 (1.05-1.20) | 1.21 (1.13-1.29) | 1.43 (1.34-1.52) | <0.001 |
| **CVD-specific mortality** |  |  |  |  |  |
| Events (n, %) | 706 (0.7) | 1109 (1.1) | 1333 (1.3) | 2403 (2.5) | <0.001 |
| Crude HR (95% CI) | 1.00 | 1.60 (1.45-1.76) | 1.95 (1.78-2.14) | 3.67 (3.37-3.99) | <0.001 |
| Adjusted HR (95% CI) | 1.00 | 1.30 (1.17-1.44) | 1.54 (1.40-1.71) | 2.68 (2.44-2.93) | <0.001 |

Note: Adjusted for age, sex, race/ethnicity, BMI, education level, alcohol use, smoking status, income level, hypertension, and diabetes.

**Table S.5 Sensitivity analyses of the associations between IBI and mortality after excluding participants with CRP >10 mg/L in the UK Biobank**

|  | **IBI score (quartiles)** | | | | ***P* trend** |
| --- | --- | --- | --- | --- | --- |
|  | **Q1** | **Q2** | **Q3** | **Q4** |  |
| **UKB Participants** | 113,843 (26.1) | 113,877 (26.1) | 113,804 (26.1) | 95,102 (21.8) |  |
| **All-cause mortality** |  |  |  |  |  |
| Events (n, %) | 5227 (4.6) | 6752 (5.9) | 8438 (7.4) | 10477 (11.0) | <0.001 |
| Crude HR (95% CI) | 1.00 | 1.30 (1.25-1.35) | 1.64 (1.58-1.69) | 2.48 (2.40-2.56) | <0.001 |
| Adjusted HR (95% CI) | 1.00 | 1.15 (1.11-1.20) | 1.37 (1.31-1.42) | 1.92 (1.84-2.00) | <0.001 |
| **Cancer-specific mortality** |  |  |  |  |  |
| Events (n, %) | 2812 (2.5) | 3559 (3.1) | 4399 (3.9) | 4978 (5.2) | <0.001 |
| Crude HR (95% CI) | 1.00 | 1.27 (1.21-1.34) | 1.58 (1.51-1.66) | 2.18 (2.09-2.29) | <0.001 |
| Adjusted HR (95% CI) | 1.00 | 1.17 (1.11-1.24) | 1.38 (1.31-1.45) | 1.81 (1.71-1.91) | <0.001 |
| **CVD-specific mortality** |  |  |  |  |  |
| Events (n, %) | 864 (0.8) | 1354 (1.2) | 1685 (1.5) | 2412 (2.5) | <0.001 |
| Crude HR (95% CI) | 1.00 | 1.57 (1.45-1.71) | 1.96 (1.82-2.14) | 3.45 (3.19-3.74) | <0.001 |
| Adjusted HR (95% CI) | 1.00 | 1.26 (1.15-1.39) | 1.48 (1.35-1.63) | 2.33 (2.13-2.55) | <0.001 |

Note: Multivariable models were adjusted for age, sex, race/ethnicity, BMI, education level, alcohol use, smoking status, income level, hypertension, and diabetes.

**Table S.6 Interaction between IBI and cancer status for mortality outcomes in the UK Biobank**

| **Cohort** | **Outcome** | **χ² (df = 3)** | ***P* for interaction** |
| --- | --- | --- | --- |
| UK Biobank | All-cause mortality | 253.31 | <0.001 |
|  | Cancer-specific mortality | 47.49 | <0.001 |
|  | CVD-specific mortality | 8.62 | 0.035 |

Note: *P* for interaction was derived from the joint test of multiplicative interaction terms between IBI quartiles and cancer status in fully adjusted Cox proportional hazards models. Models were adjusted for age, sex, race/ethnicity, BMI, education level, alcohol use, smoking status, income level, hypertension, and diabetes.

**Table S.7 Continuous analyses of the associations between IBI and mortality outcomes in the UK Biobank**

| **Cohort** | **Outcome** | **Crude HR (95% CI)** | **Adjusted HR (95% CI)** |
| --- | --- | --- | --- |
| **UK Biobank** | All-cause mortality | 1.48 (1.46–1.49) | 1.36 (1.35–1.38) |
|  | Cancer-specific mortality | 1.38 (1.36–1.40) | 1.29 (1.27–1.31) |
|  | CVD-specific mortality | 1.61 (1.58–1.65) | 1.47 (1.44–1.51) |

## Note: IBI was natural log-transformed and standardized, and HRs were estimated per 1-SD increase in ln-transformed IBI. Multivariable models were adjusted for age, sex, race/ethnicity, BMI, education level, alcohol use, smoking status, income level, hypertension, and diabetes.

**Table S.8** **Sensitivity analyses of the associations between IBI and mortality after excluding deaths occurring within the first 12 months of follow-up in NHANES**

|  | **IBI score (quartiles)** | | | | ***P* trend** |
| --- | --- | --- | --- | --- | --- |
|  | **Q1** | **Q2** | **Q3** | **Q4** |  |
| **Participants, (n, weighted %)** | 5467 (25.1) | 5881 (25.0) | 6230 (25.1) | 6911 (24.8) |  |
| **All-cause mortality** |  |  |  |  |  |
| Events (n, weighted %) | 636 (7.7) | 1056 (12.6) | 1369 (16.2) | 1783 (20.9) | <0.001 |
| Crude HR (95% CI) | 1.00 | 1.65 (1.46-1.87) | 2.16 (1.92-2.43) | 2.86 (2.54-3.21) | <0.001 |
| Adjusted HR (95% CI) | 1.00 | 1.26 (1.09-1.45) | 1.60 (1.39-1.84) | 2.05 (1.77-2.36) | <0.001 |
| **Cancer-specific mortality** |  |  |  |  |  |
| Events (n, weighted %) | 171 (2.2) | 229 (2.9) | 323 (4.4) | 381 (4.5) | <0.001 |
| Crude HR (95% CI) | 1.00 | 1.31 (1.01-1.68) | 2.00 (1.46-2.86) | 2.06 (1.63-2.59) | <0.001 |
| Adjusted HR (95% CI) | 1.00 | 1.06 (0.79-1.43) | 1.54 (1.15-2.05) | 1.55 (1.16-2.07) | <0.001 |
| **CVD-specific mortality** |  |  |  |  |  |
| Events (n, weighted %) | 185 (2.3) | 324 (3.5) | 439 (4.9) | 549 (6.0) | <0.001 |
| Crude HR (95% CI) | 1.00 | 1.52 (1.22-1.90) | 2.14 (1.73-2.65) | 2.60 (2.12-3.19) | <0.001 |
| Adjusted HR (95% CI) | 1.00 | 1.13 (0.87-1.48) | 1.48 (1.14-1.90) | 1.62 (1.26-2.08) | <0.001 |
| **Participants with a history of cancer (n, weighted %)** | 357 (19.6) | 474 (23.1) | 592 (27.3) | 697 (30.0) |  |
| **All-cause mortality** |  |  |  |  |  |
| Events (n, weighted %) | 116 (21.3) | 188 (30.2) | 287 (35.3) | 386 (30.1) | <0.001 |
| Crude HR (95% CI) | 1.00 | 1.48 (1.11-1.97) | 2.00 (1.52-2.62) | 2.70 (2.09-3.05) | <0.001 |
| Adjusted HR (95% CI) | 1.00 | 1.26 (0.94-1.68) | 1.71 (1.30-2.25) | 2.24 (1.71-2.94) | <0.001 |
| **Cancer-specific mortality** |  |  |  |  |  |
| Events (n, weighted %) | 37 (6.8) | 44 (6.9) | 84 (12.2) | 120 (14.3) | <0.001 |
| Crude HR (95% CI) | 1.00 | 1.00 (0.60-1.68) | 1.81 (1.14-2.86) | 2.17 (1.40-3.37) | <0.001 |
| Adjusted HR (95% CI) | 1.00 | 1.03 (0.57-1.87) | 1.77 (1.03-3.04) | 2.25 (1.32-3.82) | 0.002 |
| **CVD-specific mortality** |  |  |  |  |  |
| Events (n, weighted %) | 25 (4.9) | 63 (9.6) | 78 (10.1) | 94 (11.4) | <0.001 |
| Crude HR (95% CI) | 1.00 | 1.98 (1.17-3.35) | 2.06 (1.23-3.44) | 2.34 (1.41-3.86) | <0.001 |
| Adjusted HR (95% CI) | 1.00 | 1.65 (0.95-2.87) | 1.72 (1.00-2.94) | 1.69 (0.98-2.91) | 0.106 |
| **Participants without a history of cancer (n, weighted %)** | 5110 (25.6) | 5407 (25.2) | 5638 (24.9) | 6214 (24.4) |  |
| **All-cause mortality** |  |  |  |  |  |
| Events (n, weighted %) | 520 (6.7) | 868 (11.1) | 1082 (14.0) | 1397 (17.8) | <0.001 |
| Crude HR (95% CI) | 1.00 | 1.66 (1.44-1.91) | 2.10 (1.84-2.41) | 2.73 (2.40-3.12) | <0.001 |
| Adjusted HR (95% CI) | 1.00 | 1.24 (1.05-1.47) | 1.54 (1.31-1.81) | 1.93 (1.64-2.28) | <0.001 |
| **Cancer-specific mortality** |  |  |  |  |  |
| Events (n, weighted %) | 134 (1.8) | 185 (2.5) | 239 (3.6) | 261 (3.4) | <0.001 |
| Crude HR (95% CI) | 1.00 | 1.35 (1.01-1.81) | 1.92 (1.46-2.53) | 1.81 (1.38-2.38) | <0.001 |
| Adjusted HR (95% CI) | 1.00 | 1.06 (0.75-1.49) | 1.44 (1.03-2.01) | 1.30 (0.92-1.84) | 0.022 |
| **CVD-specific mortality** |  |  |  |  |  |
| Events (n, weighted %) | 160 (2.1) | 261 (3.0) | 361 (4.4) | 455 (5.4) | <0.001 |
| Crude HR (95% CI) | 1.00 | 1.42 (1.11-1.82) | 2.08 (1.65-2.63) | 2.54 (2.02-3.18) | <0.001 |
| Adjusted HR (95% CI) | 1.00 | 1.02 (0.75-1.37) | 1.40 (1.05-1.86) | 1.58 (1.19-2.10) | <0.001 |

Note: Adjusted for age, sex, race/ethnicity, BMI, education level, alcohol use, smoking status, income level, hypertension, diabetes, and marital status.

**Table S.9** **Sensitivity analyses of the associations between IBI and mortality after excluding deaths occurring within the first 24 months of follow-up in NHANES**

|  | **IBI score (quartiles)** | | | | ***P* trend** |
| --- | --- | --- | --- | --- | --- |
|  | **Q1** | **Q2** | **Q3** | **Q4** |  |
| **Participants, (n, weighted %)** | 5439 (25.2) | 5834 (25.1) | 6161 (25.1) | 6779 (24.6) |  |
| **All-cause mortality** |  |  |  |  |  |
| Events (n, weighted %) | 608 (7.4) | 1009 (12.1) | 1300 (15.6) | 1651 (19.8) | <0.001 |
| Crude HR (95% CI) | 1.00 | 1.65 (1.45-1.87) | 2.16 (1.92-2.43) | 2.78 (2.47-3.13) | <0.001 |
| Adjusted HR (95% CI) | 1.00 | 1.23 (1.06-1.42) | 1.52 (1.31-1.76) | 1.92 (1.66-2.23) | <0.001 |
| **Cancer-specific mortality** |  |  |  |  |  |
| Events (n, weighted %) | 166 (2.1) | 216 (2.8) | 303 (4.2) | 343 (4.1) | <0.001 |
| Crude HR (95% CI) | 1.00 | 1.34 (1.03-1.73) | 2.04 (1.60-2.59) | 2.07 (1.63-2.62) | <0.001 |
| Adjusted HR (95% CI) | 1.00 | 0.92 (0.72-1.34) | 1.43 (1.07-1.93) | 1.48 (1.10-2.00) | 0.001 |
| **CVD-specific mortality** |  |  |  |  |  |
| Events (n, weighted %) | 173 (2.2) | 310 (3.4) | 421 (4.8) | 507 (5.7) | <0.001 |
| Crude HR (95% CI) | 1.00 | 1.57 (1.25-1.97) | 2.24 (1.80-2.80) | 2.73 (2.20-3.38) | <0.001 |
| Adjusted HR (95% CI) | 1.00 | 1.17 (0.88-1.56) | 1.63 (1.24-2.15) | 1.91 (1.45-2.51) | <0.001 |
| **Participants with a history of cancer (n, weighted %)** | 353 (19.9) | 467 (23.3) | 573 (27.2) | 658 (29.5) |  |
| **All-cause mortality** |  |  |  |  |  |
| Events (n, weighted %) | 112 (20.8) | 181 (29.4) | 268 (36.9) | 347 (45.9) | <0.001 |
| Crude HR (95% CI) | 1.00 | 1.47 (1.10-1.97) | 1.93 (1.46-2.55) | 2.57 (1.97-3.36) | <0.001 |
| Adjusted HR (95% CI) | 1.00 | 1.17 (0.88-1.59) | 1.54 (1.16-2.05) | 2.00 (1.51-2.65) | 0.001 |
| **Cancer-specific mortality** |  |  |  |  |  |
| Events (n, weighted %) | 34 (6.4) | 42 (6.8) | 78 (11.8) | 103 (13.0) | <0.001 |
| Crude HR (95% CI) | 1.00 | 1.11 (0.65-1.89) | 2.00 (1.24-3.24) | 2.32 (1.46-3.70) | <0.001 |
| Adjusted HR (95% CI) | 1.00 | 1.03 (0.56-1.93) | 1.75 (1.00-3.09) | 2.06 (1.17-3.62) | 0.001 |
| **CVD-specific mortality** |  |  |  |  |  |
| Events (n, weighted %) | 24 (4.8) | 62 (9.3) | 73 (9.5) | 87 (11.2) | <0.001 |
| Crude HR (95% CI) | 1.00 | 2.03 (1.18-3.48) | 2.18 (1.28-3.70) | 2.74 (1.64-4.62) | <0.001 |
| Adjusted HR (95% CI) | 1.00 | 1.61 (0.90-2.89) | 1.93 (1.10-3.41) | 2.23 (1.26-3.97) | 0.004 |
| **Participants without a history of cancer (n, weighted %)** | 5086 (25.6) | 5367 (25.2) | 5588 (24.9) | 6121 (24.3) |  |
| **All-cause mortality** |  |  |  |  |  |
| Events (n, weighted %) | 496 (6.5) | 828 (10.8) | 1032 (13.5) | 1304 (17.0) | <0.001 |
| Crude HR (95% CI) | 1.00 | 1.66 (1.44-1.91) | 2.11 (1.84-2.42) | 2.68 (2.35-3.07) | <0.001 |
| Adjusted HR (95% CI) | 1.00 | 1.23 (1.03-1.46) | 1.48 (1.24-1.76) | 1.86 (1.56-2.21) | <0.001 |
| **Cancer-specific mortality** |  |  |  |  |  |
| Events (n, weighted %) | 132 (1.8) | 174 (2.4) | 225 (3.4) | 240 (3.2) | <0.001 |
| Crude HR (95% CI) | 1.00 | 1.37 (1.02-1.83) | 1.93 (1.46-2.56) | 1.83 (1.38-2.42) | <0.001 |
| Adjusted HR (95% CI) | 1.00 | 0.96 (0.67-1.37) | 1.31 (0.93-1.85) | 1.27 (0.89-1.82) | 0.057 |
| **CVD-specific mortality** |  |  |  |  |  |
| Events (n, weighted %) | 149 (2.0) | 248 (2.9) | 348 (4.3) | 420 (5.1) | <0.001 |
| Crude HR (95% CI) | 1.00 | 1.46 (1.13-1.87) | 2.20 (1.73-2.79) | 2.62 (2.07-3.32) | <0.001 |
| Adjusted HR (95% CI) | 1.00 | 1.08 (0.78-1.49) | 1.55 (1.14-2.12) | 1.81 (1.33-2.47) | <0.001 |

Note: Adjusted for age, sex, race/ethnicity, BMI, education level, alcohol use, smoking status, income level, hypertension, diabetes, and marital status.

**Table S.10 Sensitivity analyses of the associations between IBI and mortality after excluding participants with CRP >10 mg/L in NHANES**

|  | **IBI score (quartiles)** | | | | ***P* trend** |
| --- | --- | --- | --- | --- | --- |
|  | **Q1** | **Q2** | **Q3** | **Q4** |  |
| **NHANES Participants** | 5,486 (27.5) | 5,913 (27.5) | 6,257 (27.5) | 4,419 (17.5) |  |
| **All-cause mortality** |  |  |  |  |  |
| Events (n, %) | 651 (7.8) | 1082 (12.8) | 1403 (16.4) | 1178 (21.3) | <0.001 |
| Crude HR (95% CI) | 1.00 | 1.64 (1.45–1.86) | 2.15 (1.91–2.42) | 2.86 (2.53–3.24) | <0.001 |
| Adjusted HR (95% CI) | 1.00 | 1.22 (1.05–1.41) | 1.53 (1.33–1.77) | 1.90 (1.62–2.21) | <0.001 |
| **Cancer-specific mortality** |  |  |  |  |  |
| Events (n, %) | 172 (2.2) | 230 (2.8) | 331 (4.4) | 254 (4.8) | <0.001 |
| Crude HR (95% CI) | 1.00 | 1.32 (1.02–1.70) | 2.09 (1.65–2.65) | 2.33 (1.81–2.99) | <0.001 |
| Adjusted HR (95% CI) | 1.00 | 0.95 (0.70–1.30) | 1.45 (1.08–1.94) | 1.57 (1.14–2.15) | <0.001 |
| **CVD-specific mortality** |  |  |  |  |  |
| Events (n, %) | 190 (2.4) | 335 (3.6) | 453 (5.1) | 365 (6.2) | <0.001 |
| Crude HR (95% CI) | 1.00 | 1.56 (1.25–1.94) | 2.22 (1.80–2.73) | 2.76 (2.22–3.43) | <0.001 |
| Adjusted HR (95% CI) | 1.00 | 1.17 (0.89–1.54) | 1.62 (1.25–2.12) | 1.82 (1.38–2.40) | <0.001 |

Note: Multivariable models were adjusted for age, sex, race/ethnicity, BMI, education level, alcohol use, smoking status, income level, hypertension, diabetes, and marital status.

**Table S.11 Interaction between IBI and cancer status for mortality outcomes in NHANES**

| **Cohort** | **Outcome** | **χ² (df = 3)** | ***P* for interaction** |
| --- | --- | --- | --- |
| NHANES | All-cause mortality | 0.76 | 0.859 |
|  | Cancer-specific mortality | 4.61 | 0.203 |
|  | CVD-specific mortality | 2.51 | 0.473 |

Note: *P* for interaction was derived from the joint test of multiplicative interaction terms between IBI quartiles and cancer status in fully adjusted Cox proportional hazards models. Models were adjusted for age, sex, race/ethnicity, BMI, education level, alcohol use, smoking status, income level, hypertension, diabetes, and marital status.

**Table S.12 Continuous analyses of the associations between IBI and mortality outcomes in NHANES**

| **Cohort** | **Outcome** | **Crude HR (95% CI)** | **Adjusted HR (95% CI)** |
| --- | --- | --- | --- |
| **NHANES** | All-cause mortality | 1.50 (1.45–1.56) | 1.37 (1.31–1.44) |
|  | Cancer-specific mortality | 1.39 (1.30–1.49) | 1.28 (1.16–1.41) |
|  | CVD-specific mortality | 1.51 (1.42–1.61) | 1.38 (1.26–1.51) |

## Note: IBI was natural log-transformed and standardized, and HRs were estimated per 1-SD increase in ln-transformed IBI. Multivariable models were adjusted for age, sex, race/ethnicity, BMI, education level, alcohol use, smoking status, income level, hypertension, diabetes, and marital status.

**Table S.13 General characteristics of participants with digestive tract cancers in the UK Biobank (N = 9688)**

| **Characteristic** | **N** | **%** |
| --- | --- | --- |
| **Age (years, n, %)** |  |  |
| <65 | 7027 | 72.5 |
| ≥65 | 2661 | 27.5 |
| **Sex (n, %)** |  |  |
| Male | 5782 | 59.7 |
| Female | 3906 | 40.3 |
| **Race/ethnicity (n, %)** |  |  |
| White | 9313 | 96.1 |
| Non-white | 375 | 3.9 |
| **Education (n, %)** |  |  |
| <High school | 2284 | 23.9 |
| ≥High school | 7282 | 76.1 |
| **Income level (n, %)** |  |  |
| Low | 2456 | 30.0 |
| Middle | 5385 | 66.0 |
| High | 323 | 4.0 |
| **BMI (kg/m^2^, n, %)** |  |  |
| ≤24.9 | 2498 | 26.3 |
| 25-29.9 | 4247 | 44.7 |
| ≥30.0 | 2759 | 29.0 |
| **Alcohol use (n, %)** |  |  |
| No | 737 | 7.6 |
| Yes | 8946 | 92.4 |
| **Tobacco use (n, %)** |  |  |
| No | 5298 | 54.9 |
| Yes | 4354 | 45.1 |
| **Hypertension (n, %)** |  |  |
| No | 4872 | 50.3 |
| Yes | 4807 | 49.7 |
| **Diabetes (n, %)** |  |  |
| No | 8726 | 90.3 |
| Yes | 936 | 9.7 |
| **CVD (n, %)** |  |  |
| No | 5779 | 59.8 |
| Yes | 3884 | 40.2 |

**Table S.14** **Distribution of participants across IBI quartiles according to digestive tract cancer status in the UK Biobank**

| **Characteristic** | **Participants with digestive tract cancers** | **Participants without digestive tract cancers** | ***P* value** |
| --- | --- | --- | --- |
| **IBI quartile** |  |  | <0.001 |
| Q1 | 1738 (17.9) | 9614 (21.3) |  |
| Q2 | 2250 (23.2) | 10625 (23.5) |  |
| Q3 | 2555 (26.4) | 11538 (25.6) |  |
| Q4 | 3145 (32.5) | 13348 (29.6) |  |

**Table S.15 Sensitivity analyses of the associations between IBI quartiles and mortality among participants with digestive tract cancers in the UK Biobank after excluding deaths occurring within the first 12 months of follow-up**

|  | **IBI score (quartiles)** | | | | ***P* trend** |
| --- | --- | --- | --- | --- | --- |
|  | **Q1** | **Q2** | **Q3** | **Q4** |  |
| **Participants, n (%)** | 1729 (18.1) | 2240 (23.4) | 2535 (26.5) | 3055 (32.0) |  |
| **All-cause mortality** |  |  |  |  |  |
| Events (n, %) | 707 (40.9) | 979 (43.7) | 1135 (44.8) | 1494 (48.9) |  |
| Crude HR (95% CI) | 1.00 | 1.11 (1.01-1.22) | 1.14 (1.03-1.25) | 1.30 (1.19-1.43) | <0.001 |
| Adjusted HR (95% CI) | 1.00 | 1.10 (0.99-1.23) | 1.09 (0.98-1.22) | 1.25 (1.12-1.38) | <0.001 |
| **Cancer-specific mortality** |  |  |  |  |  |
| Events (n, %) | 651 (37.7) | 908 (40.5) | 1030 (40.6) | 1289 (42.2) |  |
| Crude HR (95% CI) | 1.00 | 1.11 (1.01-1.23) | 1.11 (1.01-1.22) | 1.19 (1.08-1.31) | 0.001 |
| Adjusted HR (95% CI) | 1.00 | 1.13 (1.01-1.26) | 1.10 (0.98-1.22) | 1.17 (1.05-1.30) | 0.019 |
| **CVD-specific mortality** |  |  |  |  |  |
| Events (n, %) | 25 (1.4) | 27 (1.2) | 44 (1.7) | 67 (2.2) |  |
| Crude HR (95% CI) | 1.00 | 0.87 (0.50–1.49) | 1.25 (0.76–2.04) | 1.67 (1.05–2.64) | 0.004 |
| Adjusted HR (95% CI) | 1.00 | 0.61 (0.33–1.12) | 0.85 (0.49–1.46) | 1.06 (0.63–1.79) | 0.302 |

Note: Adjusted for age, sex, race/ethnicity, BMI, education level, alcohol use, smoking status, income level, hypertension, and diabetes.

**Table S.****16 Sensitivity analyses of the associations between IBI quartiles and mortality among participants with digestive tract cancers in the UK Biobank after excluding deaths occurring within the first 24 months of follow-up**

|  | **IBI score (quartiles)** | | | | ***P* trend** |
| --- | --- | --- | --- | --- | --- |
|  | Q1 | Q2 | Q3 | Q4 |  |
| **Participants, n (%)** | 1709 (18.3) | 2196 (23.5) | 2479 (26.5) | 2957 (31.7) |  |
| **All-cause mortality** |  |  |  |  |  |
| Events (n, %) | 687 (40.2) | 935 (42.6) | 1079 (43.5) | 1396 (47.2) |  |
| Crude HR (95% CI) | 1.00 | 1.09 (0.99–1.20) | 1.11 (1.01–1.22) | 1.26 (1.15–1.38) | <0.001 |
| Adjusted HR (95% CI) | 1.00 | 1.08 (0.96–1.20) | 1.05 (0.95–1.18) | 1.20 (1.08–1.33) | 0.001 |
| **Cancer-specific mortality** |  |  |  |  |  |
| Events (n, %) | 631 (36.9) | 865 (39.4) | 978 (39.5) | 1195 (40.4) |  |
| Crude HR (95% CI) | 1.00 | 1.10 (0.99–1.22) | 1.10 (0.99–1.21) | 1.17 (1.06–1.29) | 0.002 |
| Adjusted HR (95% CI) | 1.00 | 1.10 (0.98–1.24) | 1.06 (0.95–1.19) | 1.14 (1.02–1.28) | 0.049 |
| **CVD-specific mortality** |  |  |  |  |  |
| Events (n, %) | 25 (1.5) | 26 (1.2) | 41 (1.7) | 67 (2.3) |  |
| Crude HR (95% CI) | 1.00 | 0.84 (0.48–1.45) | 1.16 (0.71–1.91) | 1.67 (1.05-2.64) | 0.004 |
| Adjusted HR (95% CI) | 1.00 | 0.59 (0.32–1.09) | 0.78 (0.45–1.36) | 1.07 (0.64–1.81) | 0.269 |

Note: Adjusted for age, sex, race/ethnicity, BMI, education level, alcohol use, smoking status, income level, hypertension, and diabetes.

**Table S.17 General characteristics of participants in the gastric cancer cohort**

| **Characteristic** | **Classification** | **Frequency** |
| --- | --- | --- |
| Age (years), mean (SD) | 62.1 (10.1) |  |
| Age | <65 | 468 (59.0) |
|  | ≥65 | 325 (41.0) |
| Sex | Male | 571 (72.0) |
|  | Female | 222 (28.0) |
| Tumor size | <5cm | 499 (62.9) |
|  | ≥5cm | 294 (37.1) |
| Tumor stage | TNM I/II | 235 (29.6) |
|  | TNM III/IV | 558 (70.4) |
| Vascular invasion | Negative | 212 (26.7) |
|  | Positive | 581 (73.3) |
| Neural invasion | Negative | 284 (35.8) |
|  | Positive | 509 (64.2) |
| WHO classification | Tubular Adenocarcinoma | 730 (92.1) |
|  | Signet-ring cell carcinoma | 53 (6.7) |
|  | Others | 10 (1.3) |
| Chemotherapy | No | 463 (58.4) |
|  | Yes | 330 (41.6) |
| Histological classification | Low | 310 (39.1) |
|  | Medium | 477 (60.2) |
|  | High | 6 (0.8) |
| IBI | Q1 | 198 (25.0) |
|  | Q2 | 199 (25.0) |
|  | Q3 | 198 (25.0) |
|  | Q4 | 198 (25.0) |

**Table S.18** **Distribution of participant characteristics across IBI quartiles in the gastric cancer cohort**

| **Characteristic** | **Classification** | **IBI score (quartiles)** | | | | ***P* value** |
| --- | --- | --- | --- | --- | --- | --- |
|  |  | **Q1 (n=198)** | **Q2 (n=199)** | **Q3 (n=198)** | **Q4 (n=198)** |  |
| Age | <65 | 146 (73.7) | 128 (64.3) | 103 (52.0) | 91 (46.0) | <0.001 |
|  | ≥65 | 52 (26.3) | 71 (35.7) | 95 (48.0) | 107 (54.0) |  |
| Sex | Male | 128 (64.6) | 147 (73.9) | 143 (72.2) | 153 (77.3) | 0.039 |
|  | Female | 70 (35.4) | 52 (26.1) | 55 (27.8) | 45 (22.7) |  |
| Tumor size | <5cm | 147 (74.2) | 152 (76.4) | 108 (54.5) | 92 (46.5) | <0.001 |
|  | ≥5cm | 51 (25.8) | 47 (23.6) | 90 (45.5) | 106 (53.5) |  |
| Tumor stage | I/II | 80 (40.4) | 69 (34.7) | 41 (20.7) | 45 (22.7) | <0.001 |
|  | III/IV | 118 (59.6) | 130 (65.3) | 157 (79.3) | 153 (77.3) |  |
| Vascular invasion | Negative | 60 (30.3) | 64 (32.2) | 42 (21.2) | 46 (23.2) | 0.035 |
|  | Positive | 138 (69.7) | 135 (67.8) | 156 (78.8) | 152 (76.8) |  |
| Neural invasion | Negative | 81 (40.9) | 75 (37.7) | 67 (33.8) | 61 (30.8) | 0.169 |
|  | Positive | 117 (59.1) | 124 (62.3) | 131 (66.3) | 137 (69.2) |  |
| WHO classification | Tubular adenocarcinoma | 177 (89.4) | 178 (89.4) | 187 (94.4) | 188 (94.9) | 0.011 |
|  | Signet-ring cell carcinoma or others | 21 (10.6) | 21 (10.6) | 11 (5.6) | 10 (5.1) |  |
| Chemotherapy | No | 107 (54.0) | 119 (59.8) | 111 (56.1) | 126 (63.6) | 0.222 |
|  | Yes | 91 (46.0) | 80 (40.2) | 87 (43.9) | 72 (36.4) |  |
| Histological classification | Low | 90 (45.5) | 83 (41.7) | 65 (32.8) | 72 (36.4) | 0.050 |
|  | Medium or high | 108 (54.5) | 116 (58.3) | 133 (67.2) | 126 (63.6) |  |

**Table S.19 Sensitivity analyses of the associations between IBI and mortality after excluding participants with CRP >10 mg/L in the gastric cancer cohort**

|  | **IBI score (quartiles)** | | | | ***P* trend** |
| --- | --- | --- | --- | --- | --- |
|  | **Q1** | **Q2** | **Q3** | **Q4** |  |
| **Gastric cancer Participants** | 197 (36.2) | 196 (36.0) | 138 (25.4) | 13 (2.4) |  |
| **All-cause mortality** |  |  |  |  |  |
| Events (n, %) | 26 (13.2) | 30 (15.3) | 42 (30.4) | 6 (46.2) | <0.001 |
| Crude HR (95% CI) | 1.00 | 1.21 (0.71-2.05) | 2.05 (1.26-3.36) | 2.44 (1.00-5.95) | 0.001 |
| Adjusted HR (95% CI) | 1.00 | 1.19 (0.70-2.04) | 1.84 (1.11-3.06) | 2.50 (0.98-6.35) | 0.007 |

Note: Multivariable models were adjusted for age, sex, tumor size, TNM stage, vascular invasion, neural invasion, WHO histological classification, histological differentiation, and chemotherapy.

**Table S.20 Continuous analysis of the association between IBI and all-cause mortality in the gastric cancer cohort**

| **Cohort** | **Outcome** | **Crude HR (95% CI)** | **Adjusted HR (95% CI)** |
| --- | --- | --- | --- |
| **Gastric cancer cohort** | All-cause mortality | 1.55 (1.39–1.74) | 1.44 (1.27–1.63) |

## Note: IBI was natural log-transformed and standardized, and HRs were estimated per 1-SD increase in ln-transformed IBI. Multivariable models were adjusted for age, sex, tumor size, TNM stage, vascular invasion, neural invasion, WHO histological classification, histological differentiation, and chemotherapy.

## 2 Supplementary Figures

**Figure** **S.1. The cumulative incidence functions (CIFs) for non-cancer and non-CVD deaths as competing events, including cancer-specific and CVD-specific mortality curves in the total population (A, B), participants with a history of cancer (C, D), and participants without a history of cancer (E, F).**
